# Computational Alanine Scanning and Structural Analysis of the SARS-CoV-2 Spike Protein/Angiotensin-Converting Enzyme 2 Complex

**DOI:** 10.1021/acsnano.0c04674

**Published:** 2020-08-24

**Authors:** Erik Laurini, Domenico Marson, Suzana Aulic, Maurizio Fermeglia, Sabrina Pricl

**Affiliations:** † Molecular Biology and Nanotechnology Laboratory (MolBNL@UniTS), DEA, University of Trieste, 34127 Trieste, Italy; ‡ Department of General Biophysics, Faculty of Biology and Environmental Protection, University of Lodz, 90-136 Lodz, Poland

**Keywords:** SARS-CoV-2 spike protein, ACE2, receptor-binding domain, molecular dynamics, computational alanine-scanning mutagenesis, molecular mechanics/Poisson−Boltzmann surface area, free energy of binding

## Abstract

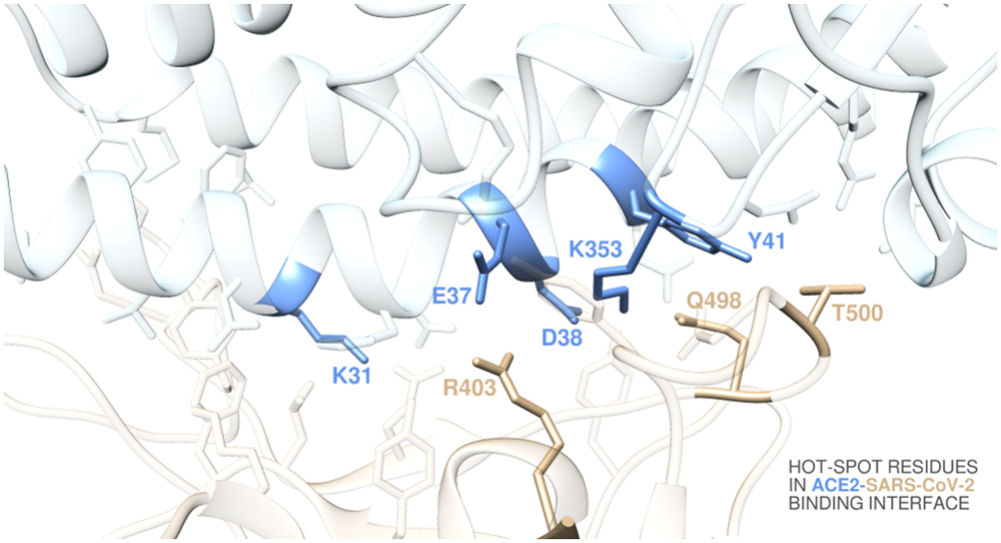

The recent emergence of the pathogen severe acute respiratory syndrome coronavirus 2 (SARS-CoV-2), the etiological agent for the coronavirus disease 2019 (COVID-19), is causing a global pandemic that poses enormous challenges to global public health and economies. SARS-CoV-2 host cell entry is mediated by the interaction of the viral transmembrane spike glycoprotein (S-protein) with the angiotensin-converting enzyme 2 gene (ACE2), an essential counter-regulatory carboxypeptidase of the renin-angiotensin hormone system that is a critical regulator of blood volume, systemic vascular resistance, and thus cardiovascular homeostasis. Accordingly, this work reports an atomistic-based, reliable *in silico* structural and energetic framework of the interactions between the receptor-binding domain of the SARS-CoV-2 S-protein and its host cellular receptor ACE2 that provides qualitative and quantitative insights into the main molecular determinants in virus/receptor recognition. In particular, residues D38, K31, E37, K353, and Y41 on ACE2 and Q498, T500, and R403 on the SARS-CoV-2 S-protein receptor-binding domain are determined as true hot spots, contributing to shaping and determining the stability of the relevant protein–protein interface. Overall, these results could be used to estimate the binding affinity of the viral protein to different allelic variants of ACE2 receptors discovered in COVID-19 patients and for the effective structure-based design and development of neutralizing antibodies, vaccines, and protein/protein inhibitors against this terrible new coronavirus.

In December 2019, a previously unidentified severe acute respiratory syndrome (SARS) coronavirus (CoV)—named SARS-CoV-2—was discovered and was isolated and sequenced by January 2020 in Wuhan, Hubei province of China.^1,2^ This virus is currently associated with an ongoing epidemic of atypical pneumonia (Coronavirus disease 19, or COVID-19) that, as of June 2, 2020, has affected almost 6,300,000 people and claimed more than 380,000 lives around the world.^3^ On March 11, 2020, the World Health Organization (WHO) declared the SARS-CoV-2 pandemic a public health emergency of international concern; as a consequence, at the time of writing well over 100 countries worldwide have just started to lift full or partial lockdowns, economically and personally affecting billions of people. Italy, home of the present authors, was one of the most affected countries in the world, with more than 233,000 cases and 33,600 deaths.^4^

CoVs are a group of large and enveloped viruses with a positive-sense, single-strand RNA genome classified into four genera (α, β, γ, and δ).^5^ Before SARS-CoV-2, only two other members of this pathogen family—also belonging to the beta genus—have crossed the species barrier to cause lethal pneumonia in humans: the severe acute respiratory syndrome coronavirus (SARS-CoV) and the Middle East respiratory syndrome coronavirus (MERS-CoV).^6^ SARS-CoV also initially emerged in China (Guangdong province) in 2002–2003 and swiftly spread *via* air-travel routes over five continents, globally infecting more than 8000 people, 10% of which with fatal *exitus*. Nearly 10 years later, MERS-CoV emerged in the Arabian Peninsula, infecting a substantially smaller number of individuals (∼2500) yet claiming 858 lives.^6^

Both SARS-CoV and MERS-CoV are zoonotic pathogens originating from animals.^7^ Whereas these two viruses were suggested to originate from bats, the reservoir host fueling their spillovers to humans was determined to be palm civets and dromedary camels, respectively. On the other hand, the source of SARS-CoV-2 is currently an issue of active debate, with bats and/or Malayan pangolins possibly serving as reservoir hosts for this new CoV.^8^ Independently of their original source, the recurrent human infections by coronavirus pathogens, including the four low pathogenicity, human-endemic HCoV-OC43, HCoV-HKU1, HCoV-NL63, and HCoV-229E,^9^ strongly suggest that future zoonotic transmission events of these large-genome viruses may continue.^10^ Unfortunately, notwithstanding this gloomy perspective, no therapeutic option or vaccines have been approved against any human-infecting coronaviruses to date.

The transmembrane spike glycoprotein (S-protein) that forms homotrimers protruding from the viral surface plays the fundamental role of assisting CoV pathogen entry into host cells. This complex process initially involves S-protein/host receptor binding followed by proteolytic processing of the viral glycoprotein to promote virus–cell membrane fusion.^11^ For SARS-CoV and SARS-CoV-2, a well-characterized S-protein region—the receptor-binding domain (S-RBD)—specifically recognizes angiotensin-converting enzyme 2 (ACE2) as its cellular receptor,^12−14^ and it is now well-established that host susceptibility to SARS-CoV/CoV-2 is primarily determined by the binding affinity of the viral S-RBD for ACE2 during the initial viral attachment step.^14−19^

To date, computer modeling of the interaction between the S-RBD of SARS-CoV-2 (S-RBD_CoV-2_) and ACE2 has identified some residues potentially involved in the interaction; however, the actual interactions have not been investigated in detail.^20^ In particular, although the structure of the S-RBD_CoV-2_ in complex with ACE2 has been repeatedly solved by different groups using both X-ray diffraction and cryo-TEM techniques,^14−16,18,19^ thus providing a comprehensive view of the interaction between the viral spike protein binding domain and its receptor, many questions still await an answer; for example, what is the relative energetic importance of the S-RBD_CoV-2_/ACE2 contacts? Are there a few hot spot residues on the viral protein or its receptor for these interactions? In this context, for instance, while we were preparing this work, Han and Kral published in this same journal an interesting *in silico* work in which they proposed some peptide inhibitors whose sequences were directly extracted from the ACE2 α-helical domain involved in S-RBD_CoV-2_ binding.^21^ These authors predicted these molecules could be efficient blockers of the viral protein–receptor interaction based on the average interaction energy between each inhibitor and the S-RBD_CoV-2_. Based on our own long-standing experience in the field of computational protein/protein and protein/ligand interactions,^22−42^ we believe that detailed structural/energetic information at each single residue would greatly improve our understanding of the binding between the spike of SARS-CoV-2 and its cellular receptor. Accordingly, here, we report the results obtained from a combined computational alanine-scanning interaction entropy method^43−46^ to compute residue-specific ACE and S-RBD_CoV-2_ binding free energy at their protein/protein interface. The data thus obtained allowed for a complete structural characterization of both intermolecular and intramolecular interaction networks contributing to S-RBD_CoV-2_/ACE2 binding under physiological solution-mimicking conditions and the identification of hot spot residues playing a major role in shaping and stabilizing the SARS-CoV-2 S-protein/receptor interface, as discussed below.

## Results and Discussion

**Figure 1:**
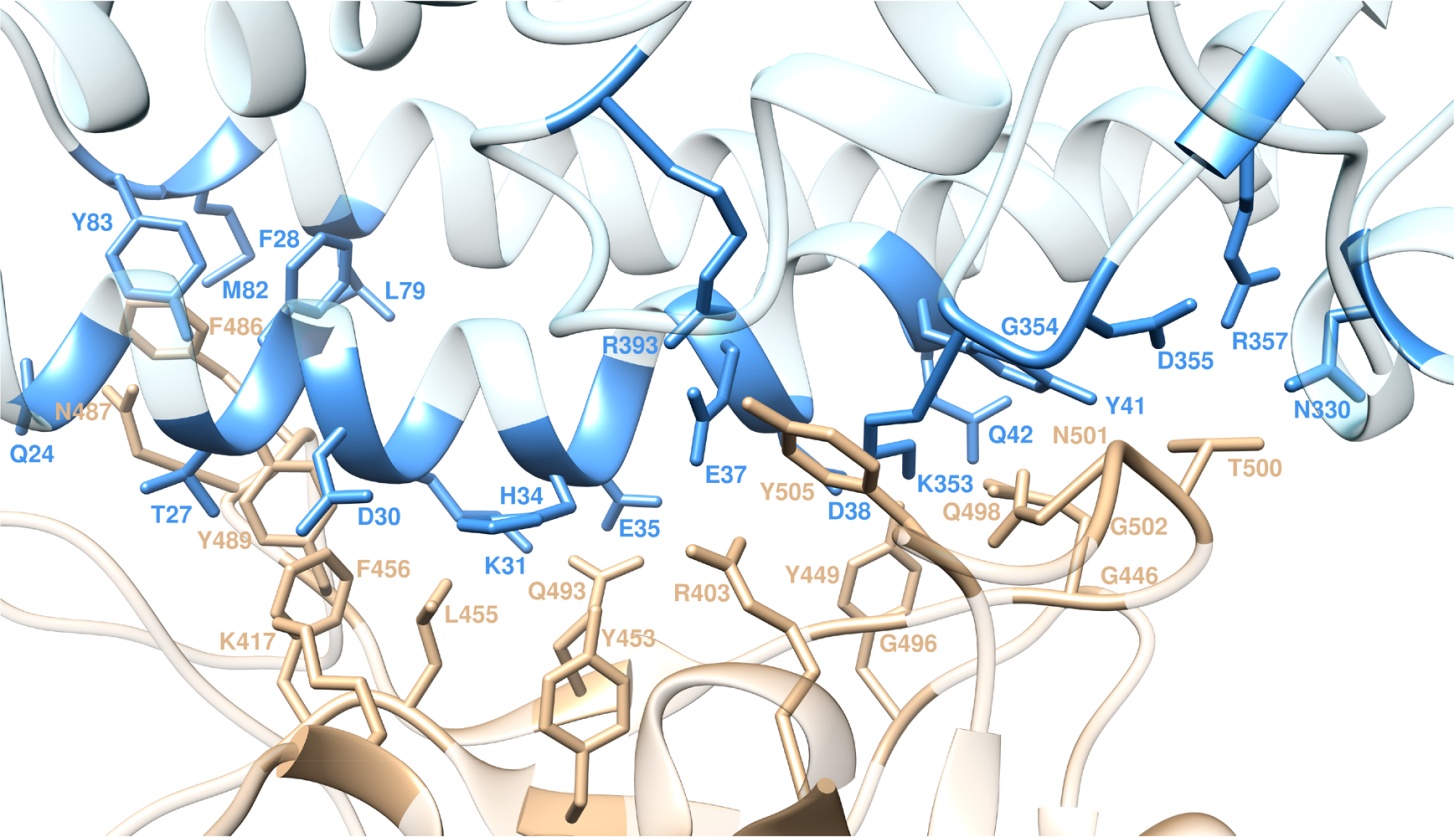
Structural details of the binding interface between ACE2 and the viral spike protein receptor-binding domain of SARS-CoV-2 (S-RBD_CoV-2_). The secondary structures of ACE2 and S-RBD_CoV-2_ are portrayed as light blue and light sienna ribbons, respectively. Each interacting protein residue is highlighted in matching-colored sticks and labeled.

According to the recent X-ray/cryo-TEM evidence, the superposition of ACE2 alone^47^ and in complex with S-RBD_CoV-2_ clearly shows that binding of the S-protein does not induce any conformational change in the relevant receptor-binding site.^14,18,19^ Moreover, the ACE2-bound S-RBD_CoV-2_ also preserves the same conformation it adopts when the full spike protein assembles into its native trimeric form.^16,17^ Briefly, the core structure of S-RBD_CoV-2_ is composed of twisted five-stranded antiparallel β-sheets (β1, β2, β3, β4, and β7), connected by short helices and flexible loops. Strands β4 and β7 are spaced by α-helices (α4 and α5), short strands (β5 and β6), and coils. This particular sequence of S-RBD_CoV-2_ domain features most of the SARS-CoV-2 residues contacting ACE2 for binding (Figure 1). On the receptor side, the N-terminal domain of ACE2 presents two lobes, the S-RBD_CoV-2_ contacting the bottom side of the smaller lobe, with a concave outer surface accommodating the N-terminal helix of the receptor (Figure 1).

Within a distance cutoff of 4.0 Å, the analysis of the equilibrated molecular dynamics (MD) trajectory (Figure S1) of ACE2 in complex with the S-protein RBD of SARS-CoV-2 shows that a total of 14 residues of S-RBD_CoV-2_ stably and effectively contact 19 residues of the receptor (Figure 1 and Tables S1 and S2). One important feature at the S-RBD_CoV-2_/ACE2 interface is the number of hydrophilic interactions detected in the relevant crystal structures,^14,18^ which are conserved in the corresponding solution simulation. Indeed, according to the present study, 14 hydrogen bonds (HBs) and two salt bridges (SBs) stably populate the S-RBD_CoV-2_/ACE2 interface (Tables S1 and S2).

**Figure 2:**
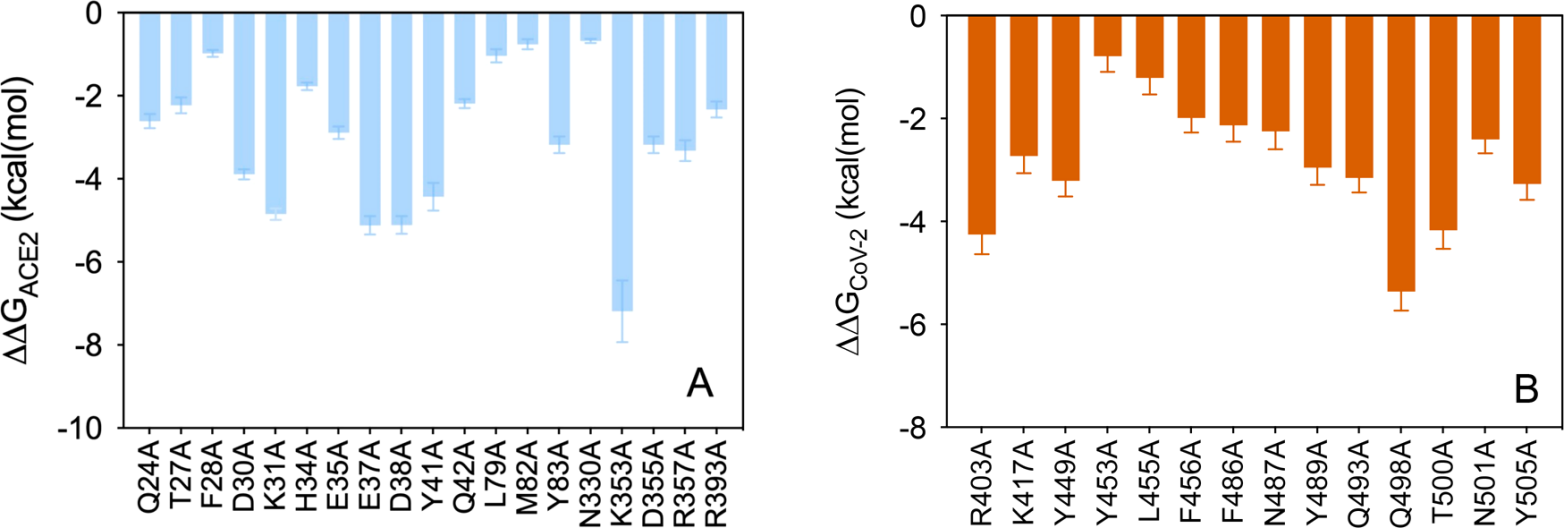
Binding energy change (ΔΔ*G* = Δ*G*_wild-type_ – Δ*G*_ALA_) obtained from the computational alanine-scanning mutagenesis for ACE2 in complex with the S-RBD_CoV-2_. (A) Mutagenesis results for the ACE2 residues at the binding interface with the viral protein RBD. (B) Mutagenesis results for the S-RBD_CoV-2_ residues at the binding interface with the receptor. Negative ΔΔ*G* values indicate unfavorable substitution for alanine in the relevant position. For the numerical values of ΔΔ*G* and all related energy terms, see the text and Tables S3 and S4.

As atomistic structural and energetic information greatly improve our understanding of the interaction between the viral S-protein RBD and its cellular ACE2 receptor, providing fundamental indications about important targets for the design of neutralizing antibodies and/or structure-based vaccine design is urgently needed in the open fight against this viral spread, with a combined description of all topical protein/protein interactions along with the corresponding energetic quantification (Figure 2 and Tables S3 and S4) being reported and discussed in detail.

### Analysis of the ACE2 Residues at the Binding Interface with the S-RBD SARS-CoV-2

#### Q24

**Figure 3:**
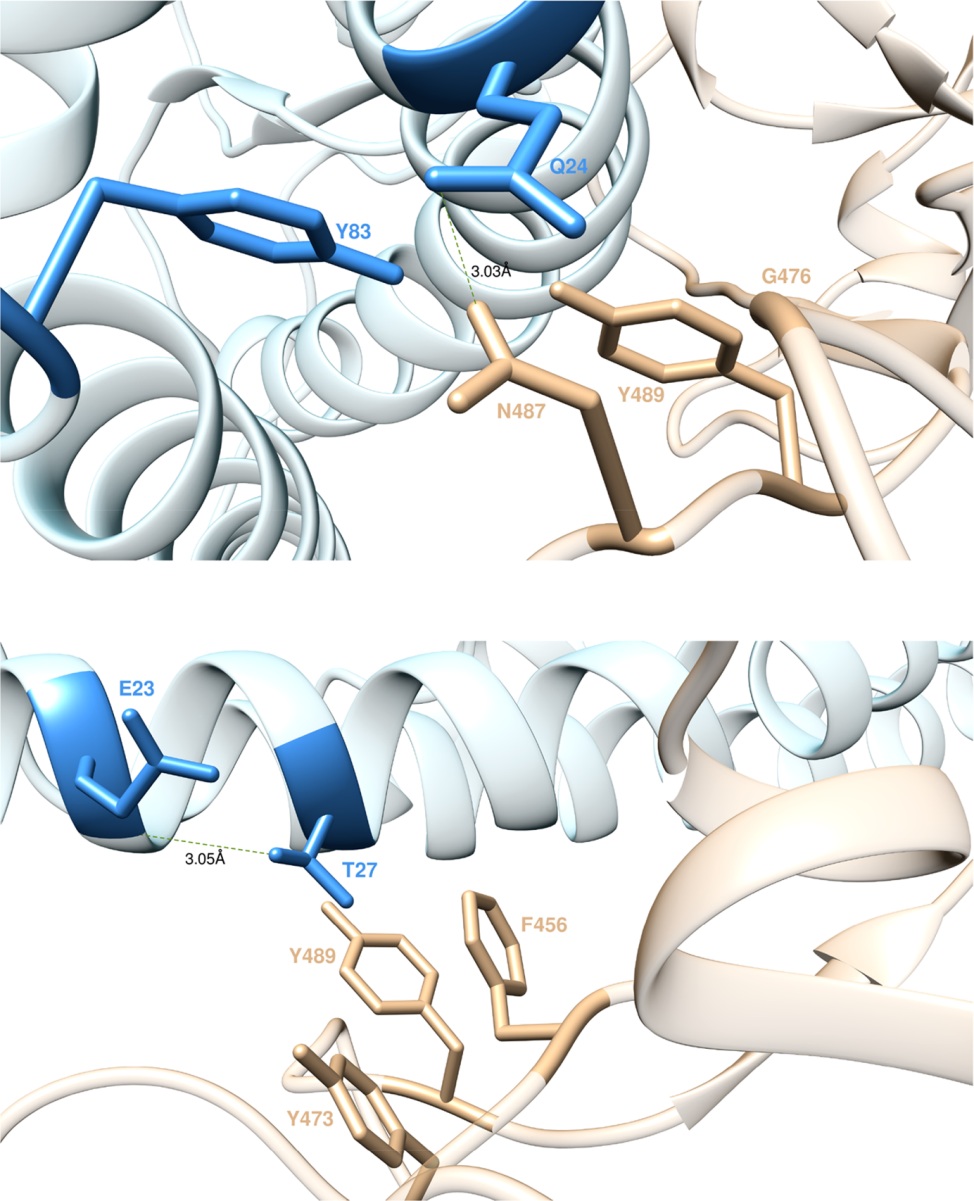
Main interactions involving ACE2 residues Q24 (top) and T27 (bottom) at the interface with S-RBD_CoV-2_ as obtained from equilibrated MD simulations. In this and all remaining figures, the secondary structures of ACE2 and S-RBD_CoV-2_ are portrayed as light blue and light sienna ribbons, respectively. Each interacting protein residue is highlighted with matching-colored sticks and labeled. Hydrogen bonds (HBs) and salt bridges (SBs) are represented as dark green and dark red broken lines, respectively, and the relevant average distances are reported accordingly (see Tables S1 and S2 for details).

ACE2 residue Q24 locates at one of the extremes of the binding interface between the receptor and the SARS-CoV-2 S-RBD; as such, any intermolecular interaction involving Q24 and the viral S-protein could be important in eventually anchoring the entire superstructure. The MD trajectory of the S-RBD_CoV-2_/ACE2 complex shows that ACE2 Q24 H-bonds the viral protein residue N487 (3.03 ± 0.18 Å) while involving two other residues G476 and Y489 in weaker contact interactions (CIs) (Figure 3, top). When ACE2 Q24 is replaced with alanine, these interface-stabilizing interactions—along with the slightly beneficial contribution from the intramolecular van der Waals contact with Y83—are no longer made, reflecting a loss of the corresponding binding free energy of ΔΔ*G*_ACE2_(Q24A) = −2.61 ± 0.17 kcal/mol (Figure 2A).

#### T27

At the interface between ACE2 and S-RBD_CoV-2_, the receptor residue T27 stabilizes the protein–protein complex *via* an internal HB with E23 (3.05 ± 0.16 Å) and through hydrophobic/van der Waals interactions with the side chains of F456, Y473, and Y489 of S-RBD_CoV-2_ (Figure 3, bottom). Replacing this receptor residue with alanine is unfavorable and produces a negative variation of the binding free energy (Figure 2A) equal to ΔΔ*G*_ACE2_(T27A) = −2.23 ± 0.19 kcal/mol.

#### Y83, M82, L79, and F28

**Figure 4:**
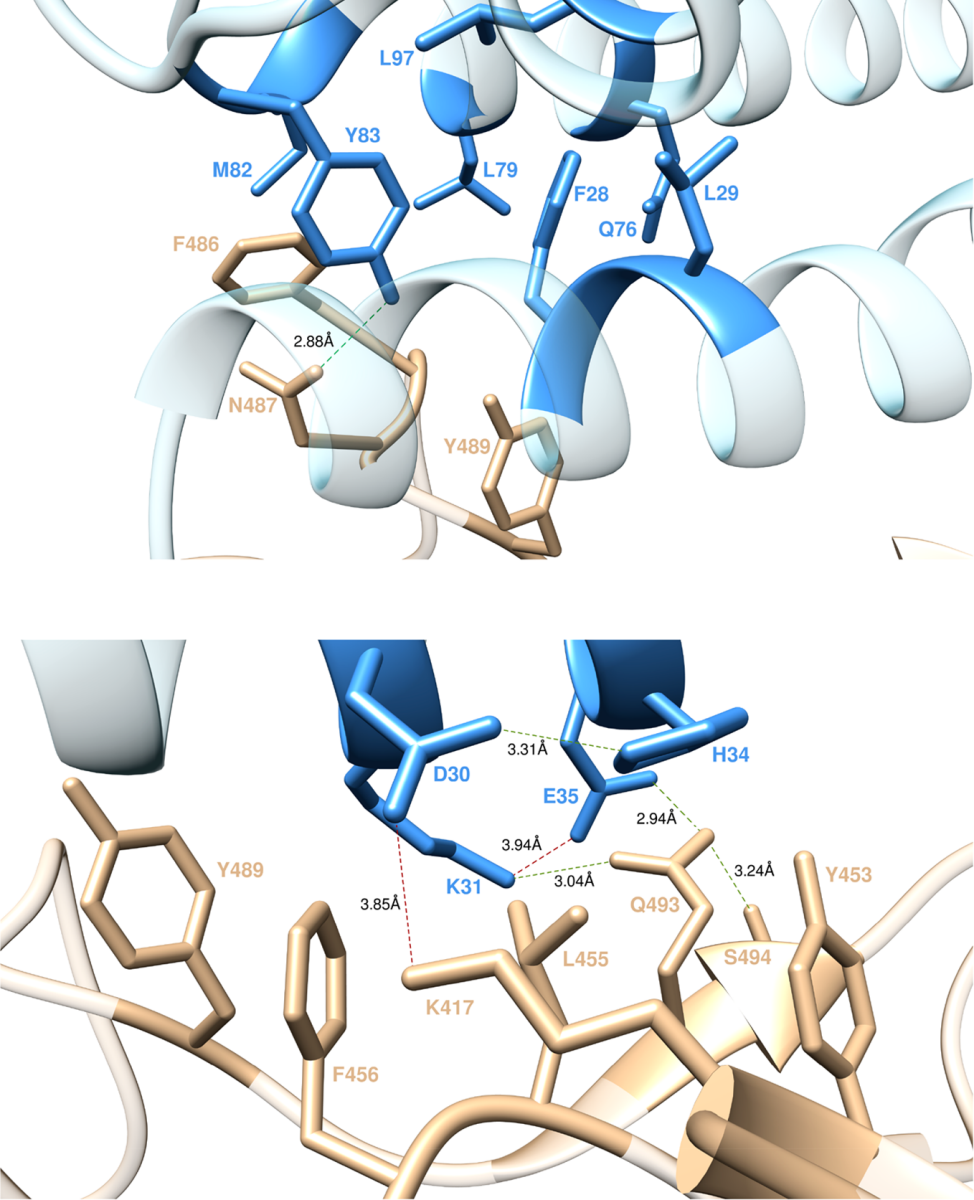
Main interactions involving ACE2 residues Y83, M82, L79, and F28 (top) and ACE2 residues D30, K31, H34, and E35 (bottom) at the interface with S-RBD_CoV-2_ as obtained from equilibrated MD simulations. Colors and other explanations as in Figure 3.

Residue Y83 of ACE2 is part of a large hydrophobic pocket surrounded by the side chains of M82, L79, L97, F28, Q76, and L29, with which it makes a number of stabilizing intramolecular CIs in the viral S-protein/receptor complex along the entire relevant MD trajectory (Figure 4, top). Moreover, Y83 exchanges a strong intermolecular HB with N487 (2.88 ± 0.17 Å), further supported by polar and dispersive CIs with Y489 and F486 (Figure 4, top). In line with these interactions, the calculated variation in binding free energy for mutating Y into A at position 83 of ACE2 is equal to ΔΔ*G*_ACE2_(Y83A) = −3.18 ± 0.20 kcal/mol (Figure 2A). In the same context, the fact that ACE2 residues F28, M82, and L79 afford only a weak network of stabilizing intra/intermolecular CIs to this interface region (Figure 4, top) is confirmed by the calculated ΔΔ*G* values obtained by changing each of these amino acids into alanine in the receptor/S-RBD_CoV-2_ complex, that is, ΔΔ*G*_ACE2_(M82A) = −0.76 ± 0.12 kcal/mol, ΔΔ*G*_ACE2_(L79A) = −1.04 ± 0.16 kcal/mol, and ΔΔ*G*_ACE2_(F28A) = −0.98 ± 0.08 kcal/mol (Figure 2A).

#### K31 and E35

In the solved crystal structure of the S-RBD_CoV-2_/ACE2 complex, K31 is seen in a strain-free, extended conformation at the protein–protein interface where it forms a stable, strong, and charge-neutralizing internal SB with E35. The MD trajectory of the corresponding complex further reveals that the K31–E35 salt bridge equilibrates at a length of 3.94 ± 0.42 Å, allowing for further stabilization *via* the formation of a strong bifurcate HB of S-RBD_CoV-2_ Q493 with both residues (3.04 ± 0.25 and 2.94 ± 0.19 Å, respectively). Also, the long side chain of K31 is involved in an extensive network of hydrophobic and van der Waals interactions, which includes the side chains of S-RBD_CoV-2_ residues L455, F456, and Y489 (Figure 4, bottom). In line, the predicted changes in binding free energy for replacing ACE2 K31 and E35 with alanine in this complex—ΔΔ*G*_ACE2_(K31A) = −4.85 ± 0.14 kcal/mol, ΔΔ*G*_ACE2_(E35A) = −2.89 ± 0.15 kcal/mol (Figure 2A)—support the prominent contribution afforded by these two residues and by K31, in particular, at the receptor/viral protein binding interface.

#### D30 and H34

ACE2 residue D30 plays an important role in shaping the relevant S-RBD_CoV-2_/receptor interface as, during the entire MD simulation, it forms an important SB with the viral protein residue K417 (3.85 ± 0.41 Å), intermolecular van der Waals interactions with F456 and L455, and a permanent intramolecular HB with ACE2 H34 (3.31 ± 0.18 Å) (Figure 4, bottom). This explains the considerable loss of binding free energy predicted upon mutating D30 into alanine within the S-RBD_CoV-2_/ACE2 complex (Figure 2A), that is, ΔΔ*G*_ACE2_(D30A) = 3.89 ± 0.12 kcal/mol. Interestingly, although ACE2 residue H34 is not directly involved in any intermolecular SB/HB with the viral protein, it nonetheless provides favorable polar/dispersive contacts with the side chains of Y453 and L455 of S-RBD_CoV-2_ (Figure 4, bottom). The absence of these CIs when H34 is mutated into alanine reflects the moderate variations of the corresponding free energy of binding (Figure 2A), that is, ΔΔ*G*_ACE2_(H34A) = −1.77 ± 0.09 kcal/mol.

#### K353, D38, and Q42

**Figure 5:**
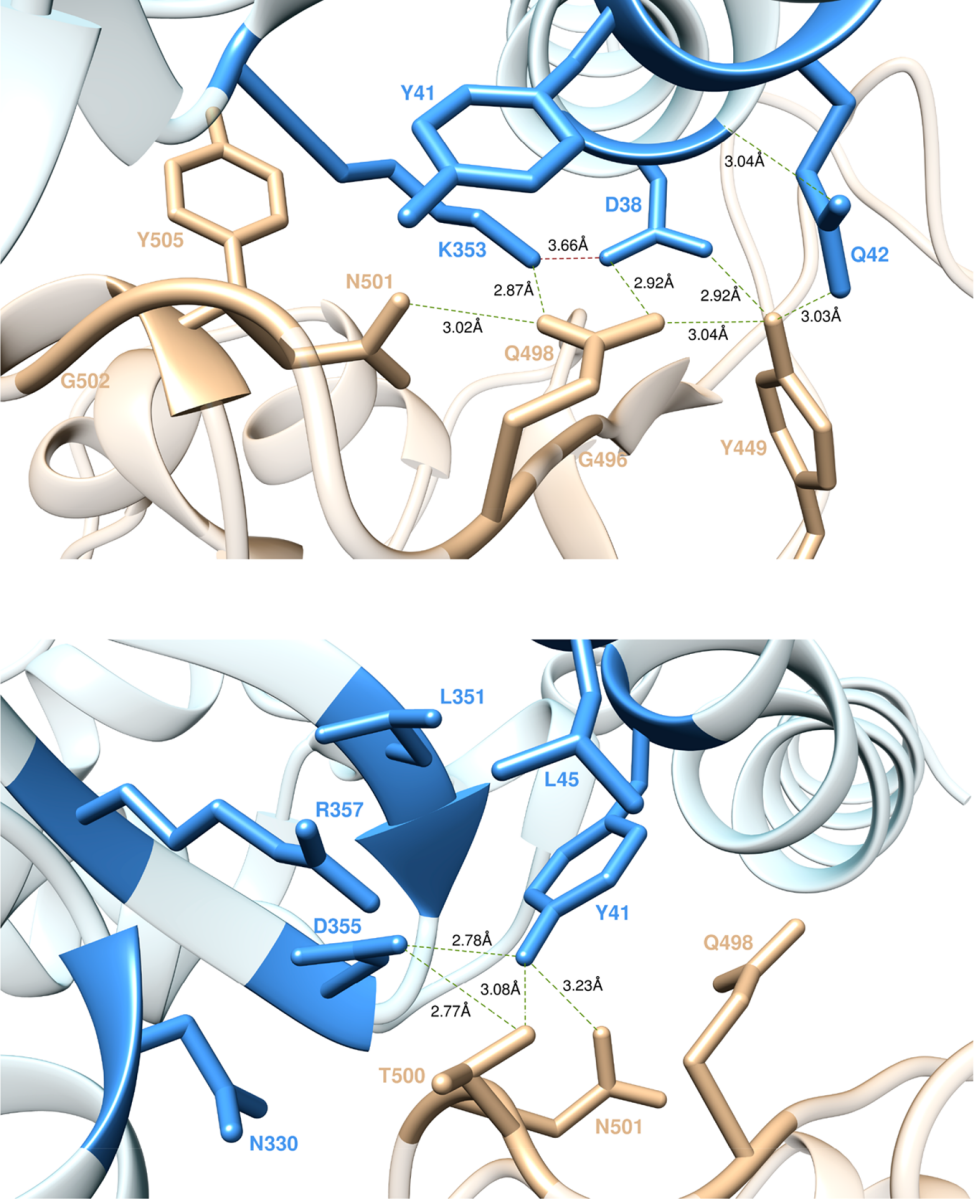
Main interactions involving ACE2 residues K353, D38, and Q42 (top) and ACE2 residues Y41 and D355 (bottom) at the interface with S-RBD_CoV-2_ as obtained from equilibrated MD simulations. Colors and other explanations as in Figure 3.

Residue K353 is an indisputable hot spot for the binding of the viral S-proteins to their human receptor. For K31, the side chain of this residue protrudes into the protein–protein interface where, by adopting a strain-free, energetically favorable conformation stabilized by an important charge-neutralizing intramolecular SB with D38 (3.38 ± 0.29 and 3.66 ± 0.39 Å, Figure 5, top), it exchanges a plethora of topical intermolecular interactions persisting through the entire time span (1 μs) of the MD simulation. In particular, the extended conformation of the K353 side chain is anchored in place by three effective intermolecular HBs—two exchanged with the backbone of G502 (2.92 ± 0.13 Å) and G496 (2.95 ± 0.21 Å) and the last with the side chain of Q498 (2.87 ± 0.13 Å)—whereas polar and van der Waals CIs with N501, Y505, and Y41 yield additional intermolecular stabilizing contacts (Figure 5, top). Removing all of these interactions by replacing the side chain of K353 with alanine results in a dramatic variation of the corresponding ΔΔ*G* value (ΔΔ*G*_ACE2_(K353A) = −7.19 ± 0.74 kcal/mol, Figure 2A), confirming the major role played by this receptor residue in binding the viral S-RBD. D38, the intramolecular SB partner of K353, is also a hot spot in the S-RBD_CoV-2_/ACE2 interaction. Indeed, as seen from the top panel of Figure 5, D38 is stably engaged in two symmetrical HBs with the side chains of the S-protein residues Q498 (2.92 ± 0.19 Å) and Y449 (2.92 ± 0.20 Å), while performing CIs with the same residue (Y449) and G496 (Figure 5, top). Additionally, D38 intramolecularly H-bonds Q42 (3.04 ± 0.18 Å), thereby favoring the formation of a stable Q42–Y449 intermolecular HB (3.03 ± 0.20 Å) and a Q42–Q498 stabilizing CI (Figure 5, top). Based on such MD-derived structural evidence, mutating D into A at position 38 of ACE2 results in the abrogation of most of these inter/intramolecular interactions; this, in turn, properly reflects the substantial variation of the corresponding binding free energy value, so that ΔΔ*G*_ACE2_(D38A) = −5.11 ± 0.21 kcal/mol (Figure 2A). Contextually, a significantly smaller ΔΔ*G* variation is predicted when replacing the side chain of Q42 with alanine—ΔΔ*G*_ACE2_(Q42A) = −2.19 ± 0.11 kcal/mol (Figure 2A)—in accord with the lesser role played by this residue at the protein–protein interface.

#### Y41, D355, and R357

**Figure 6:**
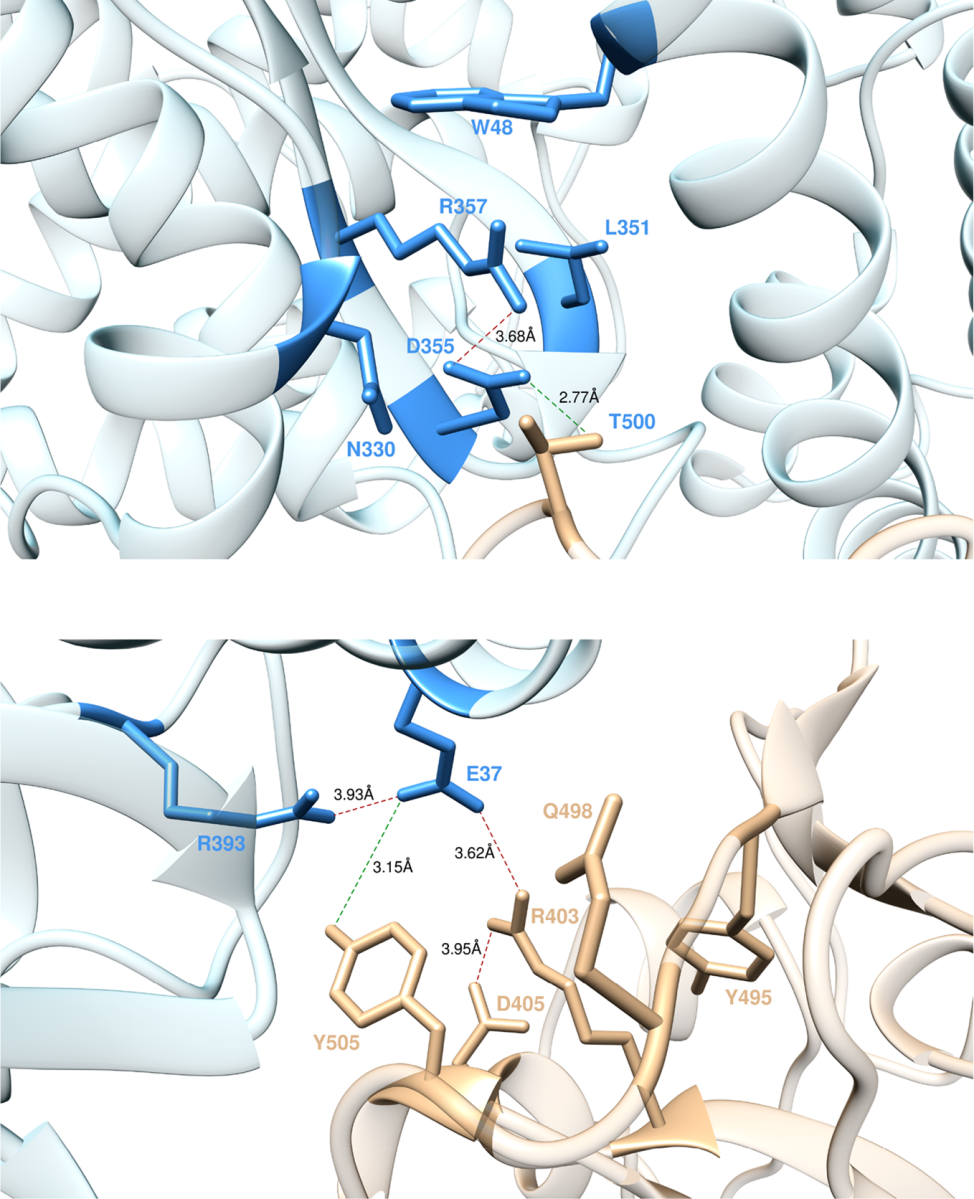
Main interactions involving ACE2 residue R357 (top) and ACE2 residues E37 and R393 (bottom) at the interface with S-RBD_CoV-2_ as obtained from equilibrated MD simulations. Colors and other explanations as in Figure 3.

According to our simulations, Y41, located on ACE2 α-helix 1, is another key residue in the interaction between the viral S-protein and its human cellular receptor. From the bottom panel of Figure 5, it can be seen that, in addition to the CI with the side chain of K353 already reported above, Y41 H-bonds D355 (2.78 ± 0.15 Å) and establishes favorable van der Waals contacts with the side chains of L45 and L351. From the intermolecular perspective, in the S-RBD_CoV-2_/ACE2 assembly, Y41 exchanges two HBs with the viral protein residues T500 (3.08 ± 0.23 Å) and N501 (3.23 ± 0.22 Å) and a CI with the side chain of Q498 (Figure 5, bottom). The corresponding value of ΔΔ*G*_ACE2_(Y41A) = −4.43 ± 0.33 kcal/mol (Figure 2A) is in line with the important contribution this residue provides to the formation of the viral protein/receptor interface. D355 is another residue playing an important role in shaping this protein/protein interface region. In addition to the main intermolecular HB with Y41 discussed above, D355 also shares the same type of interaction across the interface with T500 of SARS-CoV-2 S-RBD (2.77 ± 0.16 Å, Figure 5, bottom). Moreover, the negative charge on D355 is aptly neutralized at the complex interface *via* the formation of an internal SB with R357 (3.68 ± 0.19 Å, Figure 6, top) which, in turn, is held in place by weak CIs of polar and dispersive nature with the side chains of N330, W48, and L351 of ACE2 and of T500 of S-RBD_CoV-2_ (Figure 6, top). The relevant results of the computational alanine-scanning mutagenesis reveal a significant loss in the binding free energy when D355 is mutated to alanine—ΔΔ*G*_ACE2_(D355A) = −3.18 ± 0.20 kcal/mol, Figure 2A—as expected from the abrogation of important interactions such as HBs and SBs exquisitely involving the side chain of D355. Similarly, the replacement of R with A at ACE2 position 357 reflects a decrement of the relevant ΔΔ*G* value: ΔΔ*G*_ACE2_(R357A) = −3.32 ± 0.25 kcal/mol, Figure 2A. Finally, mutating into alanine, those other receptor residues involved in the weak interaction network just described have very little effect on protein/protein binding (*e.g.*, ΔΔ*G*_ACE2_(N330A) = −0.68 ± 0.05 kcal/mol, Figure 2A).

#### E37 and R393

According to the results of the present study, in the S-RBD_CoV-2_/ACE2 complex E37 is engaged—beside an internal SB with R393 (3.93 ± 0.38 Å)—in two important binding contacts: a HB with the side chain of Y505 (3.15 ± 0.24 Å) and a strong SB with the side chain of R403 (3.62 ± 0.39 Å, Figure 6, bottom). Thus, the E37A mutation actually shows a considerable variation in the corresponding ΔΔ*G* value—ΔΔ*G*_ACE2_(E37A) = −5.12 ± 0.22 kcal/mol, Figure 2A—making E37 another protein/protein hot spot residue. Similarly, the calculated value of ΔΔ*G* for the alanine variant of ACE2 R393 properly reflects the relevant roles played by this residue at the protein/protein interface (ΔΔ*G*_ACE2_(R393A) = −2.33 ± 0.19 kcal/mol, Figure 2A).

### Analysis of the SARS-CoV-2 S-RBD Residues at the Binding Interface with ACE2

#### Y449, Y453, and T500

According to the present MD simulation results, S-RBD_CoV-2_ residue Y449 establishes one intermolecular HB with ACE2 Q42 and one intramolecular HB with Q498 (3.04 ± 0.18 Å); also, Y449 exchanges van der Waals contacts with D38 and Q42 (Figure 5, top). The ΔΔ*G* value predicted for the corresponding Y449A replacement is therefore proportional to loss of the relevant interactions, that is, ΔΔ*G*_CoV-2_(Y449A) = −3.21 ± 0.31 kcal/mol (Figure 2B). Likewise, T500 of S-RBD_CoV-2_ establishes an extensive network of intermolecular interactions with the ACE2 receptor, which includes H-bonding with Y41 and D355, and polar/van der Waals CIs with the side chains of R357 and N330 (Figure 5, bottom, and Figure 6, top). As reported above, these interactions are instrumental in properly shaping the relevant protein/protein interface regions so that, once removed by replacing T with A on the S-RBD_CoV-2_, a significant loss in binding free energy is predicted as ΔΔ*G*_CoV-2_(T500A) = −4.17 ± 0.36 kcal/mol (Figure 2B). On the contrary, Y453 of SARS-CoV-2 plays only a minor role in the corresponding protein/protein interface. For this residue, along with the polar intermolecular interaction with ACE2 H34, only one intramolecular CI with Q493 is detected (Figure 4, bottom). Consequently, ΔΔ*G*_CoV-2_(Y453A) = −0.79 ± 0.30 kcal/mol (Figure 2B).

#### N487, Y489, and Y505

S-RBD_CoV-2_ N487 contributes to ACE2 binding *via* two HBs with Q24 and Y83 (Figures 3 and 4, top panels). In line, the alanine substitution at this S-protein position displays a variation in binding free energy of ΔΔ*G*_CoV-2_(N487A) = −2.25 ± 0.35 kcal/mol (Figure 2B). Similarly, mutating the side chain of Y489, for which only CIs with the side chains of the ACE2 residues Q24, Y83, T27, and K31 are detected along the corresponding MD trajectory (Figures 3 and 4), into alanine on the same viral protein domain results in a modest energetic variation (ΔΔ*G*_CoV-2_(Y489A) = −2.25 ± 0.35 kcal/mol, Figure 2B). Y505 of S-RBD_CoV-2_, on the other hand, participates in protein/protein hydrogen bonding to the side chain of ACE2 E37 and in CIs with R393 and K353 (Figures 5, top, and 6, bottom). Y505 also establishes a persistent internal π–cation involving the aromatic ring of this residue and the guanidinium group of R403 (Figure 6, bottom). In agreement with this interaction pattern, the Y505A mutation reduces the binding affinity of S-RBD_CoV-2_ for ACE2 by more than 3 kcal/mol (ΔΔ*G*_CoV-2_(Y505A) = −3.27 ± 0.31 kcal/mol, Figure 2B).

#### L455, F456, and F486

Mutating S-RBD_CoV-2_ L455 into alanine *in silico* does not reveal any significant change in the affinity of the relevant viral protein for the ACE2 receptor. Indeed, the side chain of this residue points to a charged pocket sealed by the side chains of ACE2 D30, K31, and H34, to which L455 provides moderately stabilizing van der Waals (L455) interactions (Figure 4, bottom). The resultant value of ΔΔ*G* is thus limited to ΔΔ*G*_CoV-2_(L455A) = −1.21 ± 0.32 kcal/mol (Figure 2B). F456 of S-RBD_CoV-2_ provides three intermolecular CIs with ACE T27, D30, and K31, an important stabilizing intramolecular π–cation interaction with the side chain of K417 (topical in assisting this lysine in salt-bridging D30), and an internal CI with Y473 (Figures 3 and 4, bottom panels). Similarly, S-RBD_CoV-2_ F486 appears to stabilize the ACE2 hydrophobic patch around Y83 by exchanging three intermolecular CIs with the receptor side chains of L79, M82, and Y83 (Figure 4, top). When all of these residues are mutated into alanine, the related values of ΔΔ*G* nicely reflect these similarities, as ΔΔ*G*_CoV-2_(F456A) = −1.99 ± 0.28 kcal/mol and ΔΔ*G*_CoV-2_(F486A) = −2.13 ± 0.32 kcal/mol (Figure 2B).

#### Q493 and Q498

At the 493 position of the SARS-CoV-2 S-protein, Q493 forms two interface-anchoring HBs with ACE2 K31 and E35 and one internal HB with the side chain of S494 (3.24 ± 0.21 Å, Figure 4, bottom). S-RBD_CoV-2_ Q498, however, affords a substantially greater number of favorable interactions to viral protein binding. As seen from Figure 5 (top panel), Q498 indeed establishes two fundamental HBs across the binding interface with ACE2 D38 and K353, along with further stabilizing CIs with the side chains of Q42 and Y41 on the receptor. Also, it exchanges an internal HB with the S-RBD_CoV-2_ N501 (3.02 ± 0.18 Å, Figure 5, bottom), in addition to the same type of interaction with Y449 discussed above; all of these contacts clearly concur in making this region one of the most structured and energetically important of the whole ACE2/S-RBD_CoV-2_ binding interface. Thus, the substitution of Q498 with alanine is accompanied by a ∼5.5 kcal/mol loss in binding free energy (ΔΔ*G*_CoV-2_(Q498A) = −5.36 ± 0.37 kcal/mol, Figure 2B), making this S-RBD_CoV-2_ residue a viral protein/receptor binding hot spot with respect to the less effective Q493, for which ΔΔ*G*_CoV-2_(Q493A) = −3.15 ± 0.29 kcal/mol (Figure 2B), in keeping with the differential contribution of these two residues to protein/protein binding.

#### N501, R403, and K417

N501 on S-RBD_CoV-2_ H-bonds the side chain of ACE2 Y41 while exchanging a polar CI with K353 and the internal HB with Q498 discussed above (Figure 5, bottom). On the other hand, R403 and K417 are the S-RBD_CoV-2_ residues making the two topical interface SBs with ACE2 E37 and D30, respectively (Figures 6 and 4, bottom panels). However, at variance with K417, R403 further establishes an internal SB with the side chain of S-RBD_CoV-2_ D405 (3.02 ± 0.18 Å, Figure 6, bottom) and two other intramolecular CIs with Y495 and Y505. Therefore, the values of the total free energy change for mutating N501, K417, and R403 in alanine—ΔΔ*G*_CoV-2_(N501A) = −2.40 ± 0.28 kcal/mol, ΔΔ*G*_CoV-2_(K417A) = −2.72 ± 0.34 kcal/mol, and ΔΔ*G*_CoV-2_(R403A) = −4.25 ± 0.39 kcal/mol (Figure 2B)—properly rank the relative importance of these residues at the protein/protein interface and flag R403 as another viral protein hot spot for receptor binding.

## Conclusions

One of the major goals of this work was to provide an atomistic-based, reliable *in silico* structural and energetic framework of the interactions between S-RBD_CoV-2_ and its host cellular receptor ACE2 that may suggest precise targets for the structure-based design and development of neutralizing antibodies, vaccines, and protein/protein inhibitors so urgently needed in the current fight against this terrible new pandemic. Accordingly, we have simulated single alanine substitutions at all different residues of ACE2 and S-RBD_CoV-2_ that form most of the protein–protein interface and estimated the variation in the corresponding free energy of binding. These mutagenesis studies provide a clear picture of the main molecular determinants in ACE2/S-RBD_CoV-2_ recognition and highlight residues D38, K31, E37, K353, and Y41 on ACE2 and Q498, T500, and R403 on the SARS-CoV-2 S-protein receptor-binding domain as true hot spots contributing to shaping and determining the stability of the relevant protein–protein interface. In addition, the results and methodologies presented and discussed above are currently being extended by our group to the estimation of the binding affinity of the viral protein to different allelic variants (AVs) of ACE2 receptors discovered in COVID-19 patients, with the ultimate goal of verifying if any of such AVs could eventually associate with different degrees of clinically observed viral pathogenicity.

## Methods

All calculations reported in this work were performed in AMBER19^48^ starting from the recent crystal structure of the ACE2/S-RBD_CoV-2_ complex (PDB ID 6M0J).^18^ The role of the protein/protein interface key residues was studied by performing a combination of molecular mechanics/Poisson–Boltzmann surface area,^49^ computational alanine scanning mutagenesis,^50^ and interaction entropy methods.^44^ All details are reported in the extended Methods section of the Supporting Information.

## Supporting Information Available

Input coordinate file (PDB)
Supplementary Tables S1–S4, Figure S1, extended Methods section, including coordinate files and force field parameters for the ACE2 Zn^2+^ binding site (PDF)

## Supplementary Material

nn0c04674_si_001.pdb
