# Supplementary material for: Computational Alanine Scanning and Structural Analysis of the SARS-CoV-2 Spike Protein/Angiotensin-Converting Enzyme 2 Complex
Source: ACS Nano. 2020 Aug 24;14(9):11821–30. doi: 10.1021/acsnano.0c04674 (PMC7448377; doi:10.1021/acsnano.0c04674)
Supplement: Supplementary file 2 — nn0c04674_si_002.pdf [file nn0c04674_si_002.pdf]

Supporting info for the paper:

Computational Alanine Scanning and Structural Analysis of the SARS-CoV-2 Spike Protein/Angiotensin-Converting Enzyme 2 Complex

Erik Laurini[1,‡], Domenico Marson[1,‡], Suzana Aulic[1], Maurizio Fermeglia[1], Sabrina Pricl[1,2*]

[1]Molecular Biology and Nanotechnology Laboratory (MolBNL@UniTS), DEA, University of Trieste, Trieste, Italy
[2]Department of General Biophysics, Faculty of Biology and Environmental Protection, University of Lodz, Lodz, Poland

Table of Contents

Table S1 Pag. 2
Table S2 Pag. 4
Table S3 Pag. 5
Table S4 Pag. 5
Figure S1 Pag. 6
Extended Methods Section Pag. 6
References Pag. 12

Table S1. Main intermolecular interactions between residues at the protein-protein interface detected during MD simulations of ACE2 in complex with the RBD of SARS-CoV-2 (COV-2). HB = hydrogen bond; SB = salt bridge; CI = contact interactions, including van der Waals/hydrophobic (vdW/h), polar (p), π/π and π/cation (π/c) interactions. In the HB column, s-s indicates side chain-side chain interactions while s-b (or b-s) and b-b indicate side chain-backbone and backbone-backbone interactions, respectively. Intermolecular interactions reported in the original crystal structure[1] and maintained in the corresponding MD simulation of the viral protein/receptor complex are shown in regular fonts while those newly reported in this work are highlighted in bold.

HB	COV-2	Length (Å)	ACE
s-s	Y449	2.92 ± 0.20	D38
**s-s**	**Q498**	**2.92 ± 0.19**	
s-s	N487	3.03 ± 0.18	Q24
s-s	N487	2.88 ± 0.17	Y83
**s-s**	**Q493**	**3.04 ± 0.25**	K31
s-s	Q493	2.94 ± 0.19	E35
s-s	Y505	3.15 ± 0.24	E37
b-b	G502	2.92 ± 0.13	K353
**s-s**	**Q498**	**2.87 ± 0.13**	
b-s	G496	2.95 ± 0.21	
s-s	T500	3.08 ± 0.23	Y41
s-s	N501	3.23 ± 0.22	
s-s	Y449	3.03± 0.20	Q42
s-s	T500	2.77 ± 0.16	D355

SB	COV2	Length (Å)
	K417	3.85 ± 0.41
	**R403**	**3.62 ± 0.39**

CI	COV-2	ACE
vdW/h	G496	D38
vdW/h	Y449	
vdW/h	G476	Q24
vdW/h	Y489	
vdW/h	F456	T27
vdW/h	Y489	
vdW/h	Y473	
p	Y489	Y83
vdW/h	F486	
vdW/h	F486	M82
vdW/h	F486	L79
vdW/h	F456	D30
vdW/h	L455	
vdW/h	Y489	K31
vdW/h	L455	
vdW/h	F456	
p	Y453	H34

vdW/h	L455	
p	Y505	R393
p	T500	R357
p	N501	
vdW/h	Y505	K353
vdW/h	**Y41**	
vdW/h	Q498	Q42
vdW/h	Q498	Y41
vdW/h	T500	N330

Table S2. Main intramolecular interactions between residues at the protein-protein interface detected during MD simulations of ACE2 in complex with the RBD of SARS-CoV-2 (COV-2). HB = hydrogen bond; SB = salt bridge; CI = contact interactions, including van der Waals/hydrophobic (vdW/h), polar (p), π/π and π/cation (π/c) interactions. In the HB column, s-s indicates side chain-side chain interactions while s-b (or b-s) and b-b indicate side chain-backbone and backbone-backbone interactions, respectively.

HB	ACE	Length (Å)	ACE	COV-2	Length (Å)	COV-2	HB
s-b	Q42	3.04 ± 0.18	D38	Y449	3.04 ± 0.18	Q498	s-s
b-s	E23	3.05 ± 0.16	T27	N501	3.02 ± 0.18		s-s
s-s	H34	3.31 ± 0.18	D30	Q493	3.24 ± 0.21	S494	s-s
s-s	D355	2.78 ± 0.15	Y41				

SB	ACE	Length (Å)	ACE	COV-2	Length (Å)	COV-2	SB
	K353	3.66 ± 0.39	D38	R403	3.95 ± 0.37	D405	
	E35	3.94 ± 0.42	K31				
	R393	3.93 ± 0.38	E37				
	R357	3.68 ± 0.19	D355				

CI	ACE	ACE	COV-2	COV-2	CI
vdW/h	L29	F28	K417	F456	π/c
vdW/h	Q76			L455	vdW/h
vdW/h	L79		R403	Y505	π/c
vdW/h	Y83			Y495	vdW/h
vdW/h	L97		Y453	Q493	vdW/h
vdW/h	Y83	Q24	F456	Y473	vdW/h
vdW/h	L79	M82			
p	N330	R357			
p	W48				
vdW/h	L351				
vdW/h	L45	Y41			
vdW/h	L351				

Table S3. Relative binding free energy and its components calculated by the combined computational alanine scanning mutagenesis – interaction entropy approach for the ACE2 residues effectively involved in the binding interface with the S-RBD of SARS-CoV-2 (see the SI Materials and Methods section for details). IE = interaction entropy. $\Delta\Delta G = \Delta G_{WT} - \Delta G_{ALA}$ (see text for details).

	**D38A**	**Q24A**	**T27A**	**F28A**	**Y83A**	**M82A**	**L79A**	**D30A**	**K31A**	**E35A**
$\Delta\Delta E_{vdW}$	-0.77	-1.96	-1.80	-1.02	-2.22	-0.72	-1.02	-0.92	-2.9	-1.27
$\Delta\Delta E_{ELE}$	-10.26	-0.99	-0.30	-0.09	-1.85	0.01	-0.03	-11.36	1.35	-6.31
$\Delta\Delta G_{SOL}$	7.18	0.75	0.14	0.16	1.07	-0.04	0.01	9.50	-3.18	5.37
$\Delta\Delta H$	-3.85	-2.20	-1.96	-0.95	-3.00	-0.75	-1.04	-2.78	-4.73	-2.21
$\Delta\Delta IE$	-1.26	-0.41	-0.27	-0.03	-0.23	-0.01	0.00	-1.11	-0.12	-0.68
**$\Delta\Delta G_{ACE2}$**	**-5.11**	**-2.61**	**-2.23**	**-0.98**	**-3.23**	**-0.76**	**-1.04**	**-3.89**	**-4.85**	**-2.89**
	**(0.38)**	**(0.37)**	**(0.39)**	**(0.28)**	**(0.40)**	**(0.32)**	**(0.36)**	**(0.32)**	**(0.34)**	**(0.35)**

	**H34A**	**R393A**	**R357A**	**E37A**	**K353A**	**Q42A**	**Y41A**	**D355A**	**N330A**
$\Delta\Delta E_{vdW}$	-1.36	-0.23	-1.29	-1.83	-2.22	-0.96	-2.43	-0.31	-0.54
$\Delta\Delta E_{ELE}$	4.37	6.67	5.82	-11.18	5.1	-1.25	-3.07	-7.89	-0.19
$\Delta\Delta G_{SOL}$	-4.24	-8.31	-7.23	8.98	-8.71	0.33	1.45	6.01	0.16
$\Delta\Delta H$	-1.23	-1.87	-2.70	-4.03	-5.83	-1.88	-4.05	-2.19	-0.57
$\Delta\Delta IE$	-0.54	-0.46	-0.62	-1.09	-1.36	-0.31	-0.38	-0.99	-0.11
**$\Delta\Delta G_{ACE2}$**	**-1.77**	**-2.33**	**-3.32**	**-5.12**	**-7.19**	**-2.19**	**-4.43**	**-3.18**	**-0.68**
	**(0.29)**	**(0.39)**	**(0.45)**	**(0.42)**	**(0.54)**	**(0.31)**	**(0.43)**	**(0.41)**	**(0.25)**

Table S4. Relative binding free energy and its components calculated by the combined computational alanine scanning mutagenesis – interaction entropy approach for the S-RBD of SARS-CoV-2 residues effectively involved in the binding interface with ACE2 (see the SI Materials and Methods section for details). IE = interaction entropy. $\Delta\Delta G = \Delta G_{WT} - \Delta G_{ALA}$ (see text for details).

	**K417A**	**Y449A**	**Y453A**	**L455A**	**F456A**	**F486A**	**N487A**	**Y489A**	**Q493A**	**Q498A**
$\Delta\Delta E_{vdW}$	-0.53	-1.45	-0.14	-1.34	-2.08	-1.59	-1.32	-2.19	-2.19	-2.95
$\Delta\Delta E_{ELE}$	-12.64	-2.43	-0.80	-0.06	-0.12	-0.19	-1.84	-1.2	-2.44	-3.37
$\Delta\Delta G_{SOL}$	11.48	0.99	0.34	0.23	0.22	-0.31	1.06	0.94	1.82	1.23
$\Delta\Delta H$	-1.69	-2.89	-0.60	-1.17	-1.98	-2.09	-2.1	-2.45	-2.81	-5.09
$\Delta\Delta IE$	-1.03	-0.32	-0.19	-0.04	-0.01	-0.04	-0.15	-0.51	-0.34	-0.27
**$\Delta\Delta G_{CoV-2}$**	**-2.72**	**-3.21**	**-0.79**	**-1.21**	**-1.99**	**-2.13**	**-2.25**	**-2.96**	**-3.15**	**-5.36**
	**(0.34)**	**(0.31)**	**(0.30)**	**(0.32)**	**(0.28)**	**(0.32)**	**(0.35)**	**(0.33)**	**(0.29)**	**(0.37)**

	**T500A**	**N501A**	**Y505A**	**R403A**
ΔΔEvdW	-2.65	-2.01	-2.06	-1.69
$\Delta\Delta E_{ELE}$	-2.26	-2.6	-2.09	6.27
$\Delta\Delta G_{SOL}$	0.99	2.39	1.29	-7.68
$\Delta\Delta H$	-3.92	-2.22	-2.86	-3.10
$\Delta\Delta IE$	-0.25	-0.18	-0.41	-1.15
**$\Delta\Delta G_{CoV-2}$**	**-4.17**	**-2.40**	**-3.27**	**-4.25**
	**(0.36)**	**(0.28)**	**(0.31)**	**(0.39)**

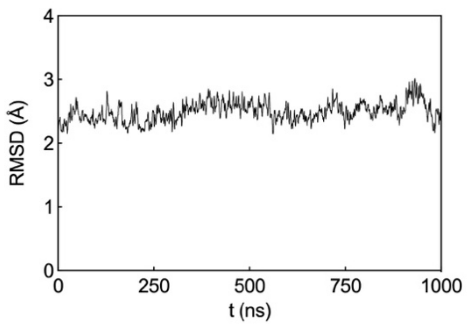

Figure S1. Root-mean-square deviation (RMSD) of the ACE2/S-RBD$_{CoV-2}$ protein complex backbone atoms as a function of MD simulation time.

Extended Methods Section

The starting structure for the wild type ACE2 protein in complex with the SARS-CoV-2 S-protein receptor binding domain (S-RBD$_{CoV-2}$) (PDB ID 6M0J)[1] was obtained from the RCSB Protein Data Bank.[2] All residues were protonated at their physiological state by the H++ server (http://biophysics.cs.vt.edu/H++).[3] The force field parameters for the Zinc atom and its protein bounded residues were obtained with the MCPB.py tools[4] provided within the AMBER19[5] suite of programs and GAMESS-US[6] software using the B3LYP/6-31G* level of theory.

The *tleap* software provided within AMBER19 was used to assign the ff14SB[7] and GLYCAM06j-1[8] forcefields to the starting protein/protein structure. The latter was next solvated in a box of TIP3PB[9] water molecules spanning at least 1.2 nm from each solute atoms. An appropriate number of Na^+ and Cl^- atoms were added to neutralize the system and mimic a physiological salt concentration (0.15 M).

While applying a weak restraint (10 kcal/mol) on the protein's backbone atoms, the simulation box was firstly energy minimized (3000 steps of steepest descent followed by 3000 steps of conjugated gradient algorithms), than heated to 150 K in 10 ps of canonical ensemble (NVT) molecular dynamics (MD), followed to another 50 ps MD simulation in the isothermal/isobaric ensemble (NPT, P = 1 atm, maintained by the Berendsen barostat[10]) to reach the target temperature of 300 K. The restraints were then gradually removed in 5 steps (-2 kcal/mol per step) of energy minimization (2000 steps of steepest descent followed by 2000 steps of conjugated gradient algorithms). The MD simulation was next carried out without restraints for further 10 ns in NPT conditions (*phase 1*); after this time interval, the MD data production run was further continued up to 1 μs, during which pressure was maintained using the Monte Carlo barostat implemented in AMBER (*phase 2*). Along the entire MD trajectory, electrostatic interactions were computed by means of the particle mesh Ewald (PME)[11] algorithm temperature was regulated by the Langevin method[12] (collision frequency of 3 ps^{-1}). The SHAKE algorithm[13] was applied to allow a 2 fs integration time step. All calculations were run with the *pmemd* module of AMBER19 running on the supercomputer Marconi100 (CINECA, Bologna, Italy) and on our CPU/GPU hybrid cluster. All images were produced by the UCSF Chimera software[14] and on Prism 8 GraphPad Prism version 8.0.0 for Mac (GraphPad Software, San Diego, California USA, www.graphpad.com).

After the first 5 ns of the *phase 2* MD trajectory, 5 ns MD data were selected to calculate enthalpy and entropy contributions. Configurational sampling was preformed accordingly, with a time step of 10 fs; thus, a total of 500 000 snapshots, sufficient for the interaction entropy (IE) calculations,[15-]

[17] were extracted from the relevant MD trajectory for the calculation of the protein/protein residue-specific interactions.
The free energy was calculated for each molecular species (protein/protein complex, ACE2, and S-RBD$_{COV-2}$) in the framework of the MM/PBSA ansatz,[18] and the protein/protein binding free energy was computed as the difference:

$$\Delta G = G_{ACE2/S\text{-}RBDCOV\text{-}2} - (G_{ACE2} + G_{S\text{-}RBDCOV\text{-}2}) = \Delta E_{vdW} + \Delta E_{ELE} + \Delta G_{SOL} - T\Delta S = \Delta H - T\Delta S \quad (1)$$

in which ΔE_{vdW} and ΔE_{ELE} represent van der Waals and electrostatic molecular mechanics energies, and ΔG_{sol} includes the solvation free energy. The internal dielectric constant was set to the values of 2, 3 and 9 for nonpolar, polar, and charged residues,[15,19] respectively. Lastly, the entropic contribution (TΔS) was explicitly computed from the MD simulation by using the Interaction Entropy (IE) method.[15-17] According to this approach, the entropic contribution to ΔG is determined from fluctuation of the interactions along the MD simulation, and IE is defined as:

$$-T\Delta S = KTln(e^{\beta\Delta E^{INT}}) \quad (2)$$

The calculation of IE by equation (2) involves the natural log of an ensemble average of $e^{\beta\Delta E^{INT}}$, which can be extracted without extra computational cost by numerical integration along the MD trajectories, as follows:

$$\langle e^{\beta\Delta E^{INT}} \rangle = \frac{1}{N} \sum_{i=1}^{N} e^{\beta\Delta E^{INT}}(t_i) \quad (3)$$

in which $\Delta E^{INT} = E^{INT} - \langle E^{INT} \rangle$ and the average interaction energy is obtained by:

$$\langle E^{INT} \rangle = \frac{1}{T} \int_0^T E^{INT}(t)dt = \frac{1}{N} \sum_{i=1}^{N} E^{INT}(t_i) \quad (4)$$

The role of the protein/protein interface key residues was studied by performing computational alanine scanning (CAS) experiments.[20] Accordingly, the absolute binding free energy of each mutant receptor - in which each key residue was replaced by alanine by truncating the mutated residue at the Cγ atom, and replacing it with a hydrogen - was calculated with the MM/PBSA method. Accordingly, the difference in the binding free energy between the wild-type (WT) protein and its alanine mutant (ALA) counterpart, ΔΔG, is given by:

$$\Delta\Delta G = \Delta G_{WILD\text{-}TYPE} - \Delta G_{ALA} \quad (5)$$

Thus, the CAS methodology allows for the estimation of the contribution of a given residue with respect to the overall protein−protein binding free energy; indeed, according to equation (5), a negative value of ΔΔG indicated a favorable contribution for the wild type residue in that position and vice versa.
At the structural level, the stability of the main protein/protein interface intermolecular and intramolecular interactions detected during the MD simulation time interval adopted for the energetic analysis was assessed along the entire duration of the MD run.

Force field parameters for the ACE2 Zn^{2+} binding site

Coordinate file for glutamic acid residue in Zn^{2+} binding site (mol2)

```
@<TRIPOS>MOLECULE
GU1
```

```
   15    14     1     0     0
SMALL
RESP Charge


@<TRIPOS>ATOM
      1 N          -21.7350   17.0320  -18.4210 N          1 GU1      -0.516300
      2 H          -22.1320   17.8590  -18.8430 H          1 GU1       0.357191
      3 CA         -21.5150   15.8580  -19.2530 CX         1 GU1       0.039700
      4 HA         -21.9560   14.9850  -18.7690 H1         1 GU1       0.149086
      5 CB         -22.2040   16.0520  -20.5960 2C         1 GU1       0.064920
      6 HB2        -22.0190   17.0550  -20.9850 HC         1 GU1       0.021908
      7 HB3        -21.7690   15.3080  -21.2500 HC         1 GU1       0.021908
      8 CG         -23.6730   15.7410  -20.6070 2C         1 GU1       0.055394
      9 HG2        -24.1890   16.4290  -19.9340 HC         1 GU1      -0.039153
     10 HG3        -24.0600   15.9040  -21.6140 HC         1 GU1      -0.039153
     11 CD         -23.9520   14.3120  -20.2020 CO         1 GU1       0.680432
     12 OE1        -24.6420   14.1190  -19.1780 Y3         1 GU1      -0.705580
     13 OE2        -23.4730   13.3850  -20.9030 O2         1 GU1      -0.673557
     14 C          -20.0270   15.5730  -19.4390 C          1 GU1       0.536600
     15 O          -19.6160   14.4090  -19.4620 O          1 GU1      -0.581900
@<TRIPOS>BOND
     1    1    2 1
     2    1    3 1
     3    3    4 1
     4    3    5 1
     5    3   14 1
     6    5    6 1
     7    5    7 1
     8    5    8 1
     9    8    9 1
    10    8   10 1
    11    8   11 1
    12   11   12 1
    13   11   13 1
    14   14   15 1
@<TRIPOS>SUBSTRUCTURE
     1 GU1         1 TEMP              0 ****  ****    0 ROOT
```

Coordinate file for first histidine residue in Zn^{2+} binding site (mol2)

```
@<TRIPOS>MOLECULE
HD1
   17    17     1     0     0
SMALL
RESP Charge


@<TRIPOS>ATOM
      1 N          -20.4740    7.0670  -14.8130 N          1 HD1      -0.415700
      2 H          -20.6500    6.1920  -15.2890 H          1 HD1       0.317305
      3 CA         -20.8800    8.3100  -15.4600 CX         1 HD1       0.018800
      4 HA         -20.0040    8.9510  -15.5570 H1         1 HD1       0.157894
      5 CB         -21.4070    7.9870  -16.8630 CT         1 HD1      -0.222931
      6 HB2        -20.6360    7.4340  -17.4030 HC         1 HD1       0.087795
      7 HB3        -22.2860    7.3580  -16.8190 HC         1 HD1       0.087795
      8 CG         -21.7920    9.1860  -17.6660 CC         1 HD1       0.027037
      9 ND1        -21.1260    9.5500  -18.8200 NA         1 HD1      -0.160885
     10 HD1        -20.3340    9.0600  -19.2160 H          1 HD1       0.313316
     11 CE1        -21.6950   10.6280  -19.3310 CR         1 HD1       0.119663
```

```
      12 HE1        -21.4030   11.1460  -20.2290 H5         1 HD1       0.214183
      13 NE2        -22.7010   10.9800  -18.5460 Y1         1 HD1      -0.508622
      14 CD2        -22.7880   10.0890  -17.5030 CV         1 HD1       0.086858
      15 HD2        -23.5370   10.0870  -16.7210 H4         1 HD1       0.119129
      16 C          -21.9280    9.0480  -14.6270 C          1 HD1       0.597300
      17 O          -21.7780   10.2390  -14.3270 O          1 HD1      -0.567900
@<TRIPOS>BOND
     1    1    2 1
     2    1    3 1
     3    3    4 1
     4    3    5 1
     5    3   16 1
     6    5    6 1
     7    5    7 1
     8    5    8 1
     9    8    9 1
    10    8   14 1
    11    9   10 1
    12    9   11 1
    13   11   12 1
    14   11   13 1
    15   13   14 1
    16   14   15 1
    17   16   17 1
@<TRIPOS>SUBSTRUCTURE
     1 HD1          1 TEMP              0 ****  ****    0 ROOT
```

Coordinate file for second histidine residue in Zn^{2+} binding site (mol2)

```
@<TRIPOS>MOLECULE
HD2
   17    17     1     0     0
SMALL
RESP Charge


@<TRIPOS>ATOM
       1 N          -21.9940   12.7620  -12.1340 N          1 HD2      -0.415700
       2 H          -22.2420   11.9150  -12.6280 H          1 HD2       0.358328
       3 CA         -23.0040   13.8090  -11.9870 CX         1 HD2       0.018800
       4 HA         -22.6170   14.7340  -12.4150 H1         1 HD2       0.065505
       5 CB         -24.2900   13.4080  -12.7110 CT         1 HD2      -0.091292
       6 HB2        -24.6720   12.4470  -12.4530 HC         1 HD2       0.032721
       7 HB3        -25.0600   14.1120  -12.3910 HC         1 HD2       0.032721
       8 CG         -24.2220   13.5440  -14.1910 CC         1 HD2       0.066406
       9 ND1        -23.5610   14.5810  -14.8120 NA         1 HD2      -0.149862
      10 HD1        -23.1230   15.3590  -14.3380 H          1 HD2       0.321043
      11 CE1        -23.6720   14.4490  -16.1200 CR         1 HD2       0.107703
      12 HE1        -23.3290   15.1500  -16.8640 H5         1 HD2       0.101460
      13 NE2        -24.3670   13.3530  -16.3670 Y2         1 HD2      -0.332596
      14 CD2        -24.7320   12.7730  -15.1790 CV         1 HD2      -0.073789
      15 HD2        -25.3210   11.8820  -15.0530 H4         1 HD2       0.135267
      16 C          -23.3080   14.0900  -10.5150 C          1 HD2       0.597300
      17 O          -23.2940   15.2470  -10.0740 O          1 HD2      -0.567900
@<TRIPOS>BOND
     1    1    2 1
     2    1    3 1
     3    3    4 1
     4    3    5 1
     5    3   16 1
```

```
      6     5     6 1
      7     5     7 1
      8     5     8 1
      9     8     9 1
     10     8    14 1
     11     9    10 1
     12     9    11 1
     13    11    12 1
     14    11    13 1
     15    13    14 1
     16    14    15 1
     17    16    17 1
@<TRIPOS>SUBSTRUCTURE
      1 HD2         1 TEMP              0 ****  ****    0 ROOT
```

Coordinate file for water molecule in Zn^{2+} binding site (mol2)

```
@<TRIPOS>MOLECULE
WT1
    3     2     1     0     0
SMALL
RESP Charge


@<TRIPOS>ATOM
       1 O         -26.6200   11.5560  -19.0380 Y4         1 WT1      -0.714686
       2 H1        -27.0800   10.7370  -19.2180 HW         1 WT1       0.443240
       3 H2        -27.3070   12.2230  -19.0410 HW         1 WT1       0.457084
@<TRIPOS>BOND
      1    1    2 1
      2    1    3 1
@<TRIPOS>SUBSTRUCTURE
      1 WT1         1 TEMP              0 ****  ****    0 ROOT
```

Coordinate file for Zn^{2+} molecule in Zn^{2+} binding site (mol2)

```
@<TRIPOS>MOLECULE
ZN1
    1     0     1     0     0
SMALL
RESP Charge


@<TRIPOS>ATOM
       1 ZN        -24.7420   12.2140  -18.2970 M1         1 ZN1       0.965715
@<TRIPOS>BOND
@<TRIPOS>SUBSTRUCTURE
      1 ZN1         1 TEMP              0 ****  ****    0 ROOT
```

Force field parameters for Zn^{2+} binding site (frcmod)

```
REMARK GOES HERE, THIS FILE IS GENERATED BY MCPB.PY
MASS
M1 65.4                             Zn ion
Y1 14.01         0.530              sp2 N in 5 memb.ring w/LP (HIS,ADE,GUA)
Y2 14.01         0.530              sp2 N in 5 memb.ring w/LP (HIS,ADE,GUA)
Y3 16.00         0.434              carboxyl and phosphate group oxygen
Y4 16.00         0.000              oxygen in TIP3P water

BOND
```

```
M1-Y4   55.6    2.0490      Created by Seminario method using MCPB.py,
replaced with an harmonic restraint (hybrid bonded/restrained non bonded
model)
Y1-M1   90.7    1.9829      Created by Seminario method using MCPB.py
Y2-M1   89.3    1.9845      Created by Seminario method using MCPB.py
Y3-M1   89.8    1.9329      Created by Seminario method using MCPB.py
CO-Y3  656.0    1.2500
CR-Y1  488.0    1.335       JCC,7,(1986),230; HIS
CR-Y2  488.0    1.335       JCC,7,(1986),230; HIS
Y1-CV  410.0    1.394       JCC,7,(1986),230; HIS
Y2-CV  410.0    1.394       JCC,7,(1986),230; HIS
Y4-HW  553.0    0.9572    ! TIP3P water

ANGL
CO-Y3-M1    65.54     114.66    Created by Seminario method using MCPB.py
CR-Y1-M1    58.24     122.34    Created by Seminario method using MCPB.py
CR-Y2-M1    31.07     119.08    Created by Seminario method using MCPB.py
M1-Y1-CV    59.96     131.01    Created by Seminario method using MCPB.py
M1-Y2-CV    31.60     134.25    Created by Seminario method using MCPB.py
M1-Y4-HW    41.44     107.64    Created by Seminario method using MCPB.py
Y1-M1-Y2    34.77     118.61    Created by Seminario method using MCPB.py
Y1-M1-Y3    27.33     115.72    Created by Seminario method using MCPB.py
Y1-M1-Y4    46.78     100.88    Created by Seminario method using MCPB.py
Y2-M1-Y3    61.02     103.02    Created by Seminario method using MCPB.py
Y2-M1-Y4    26.72     115.78    Created by Seminario method using MCPB.py
Y3-M1-Y4    54.83     102.13    Created by Seminario method using MCPB.py
2C-CO-Y3    70.0      117.00
CC-CV-Y1    70.0      120.00    AA his
CC-CV-Y2    70.0      120.00    AA his
CR-Y1-CV    70.0      117.00    AA his
CR-Y2-CV    70.0      117.00    AA his
HW-Y4-HW    100.      104.52    TIP3P water
NA-CR-Y1    70.0      120.00    AA his
NA-CR-Y2    70.0      120.00    AA his
O2-CO-Y3    80.0      126.00
Y1-CR-H5    50.0      120.00    AA his
Y1-CV-H4    50.0      120.00    AA his
Y2-CR-H5    50.0      120.00    AA his
Y2-CV-H4    50.0      120.00    AA his

DIHE
X -CR-Y1-X     2       10.0      180.0   2.0         JCC,7,(1986),230
X -CR-Y2-X     2       10.0      180.0   2.0         JCC,7,(1986),230
X -CV-Y1-X     2        4.8      180.0   2.0         JCC,7,(1986),230
X -CV-Y2-X     2        4.8      180.0   2.0         JCC,7,(1986),230
2C-2C-CO-Y3    1      0.064        0.0  -4.0
2C-2C-CO-Y3    1       0.39      180.0   2.0
2C-CO-Y3-M1    3       0.00       0.00   3.0    Treat as zero by MCPB.py
CC-CV-Y1-M1    3       0.00       0.00   3.0    Treat as zero by MCPB.py
CC-CV-Y2-M1    3       0.00       0.00   3.0    Treat as zero by MCPB.py
CO-Y3-M1-Y4    3       0.00       0.00   3.0    Treat as zero by MCPB.py
CR-Y1-M1-Y2    3       0.00       0.00   3.0    Treat as zero by MCPB.py
CR-Y1-M1-Y3    3       0.00       0.00   3.0    Treat as zero by MCPB.py
CR-Y1-M1-Y4    3       0.00       0.00   3.0    Treat as zero by MCPB.py
CR-Y2-M1-Y3    3       0.00       0.00   3.0    Treat as zero by MCPB.py
CR-Y2-M1-Y4    3       0.00       0.00   3.0    Treat as zero by MCPB.py
M1-Y1-CR-H5    3       0.00       0.00   3.0    Treat as zero by MCPB.py
M1-Y1-CV-H4    3       0.00       0.00   3.0    Treat as zero by MCPB.py
M1-Y2-CR-H5    3       0.00       0.00   3.0    Treat as zero by MCPB.py
M1-Y2-CV-H4    3       0.00       0.00   3.0    Treat as zero by MCPB.py
```

```
NA-CR-Y1-M1     3        0.00         0.00   3.0    Treat as zero by MCPB.py
NA-CR-Y2-M1     3        0.00         0.00   3.0    Treat as zero by MCPB.py
O2-CO-Y3-M1     3        0.00         0.00   3.0    Treat as zero by MCPB.py
Y1-M1-Y2-CR     3        0.00         0.00   3.0    Treat as zero by MCPB.py
Y1-M1-Y2-CV     3        0.00         0.00   3.0    Treat as zero by MCPB.py
Y1-M1-Y3-CO     3        0.00         0.00   3.0    Treat as zero by MCPB.py
Y1-M1-Y4-HW     3        0.00         0.00   3.0    Treat as zero by MCPB.py
Y2-M1-Y1-CV     3        0.00         0.00   3.0    Treat as zero by MCPB.py
Y2-M1-Y3-CO     3        0.00         0.00   3.0    Treat as zero by MCPB.py
Y2-M1-Y4-HW     3        0.00         0.00   3.0    Treat as zero by MCPB.py
Y3-CO-2C-HC     1         0.0          0.0   2.0
Y3-M1-Y1-CV     3        0.00         0.00   3.0    Treat as zero by MCPB.py
Y3-M1-Y2-CV     3        0.00         0.00   3.0    Treat as zero by MCPB.py
Y3-M1-Y4-HW     3        0.00         0.00   3.0    Treat as zero by MCPB.py
Y4-M1-Y1-CV     3        0.00         0.00   3.0    Treat as zero by MCPB.py
Y4-M1-Y2-CV     3        0.00         0.00   3.0    Treat as zero by MCPB.py

IMPR
X -O2-CO-Y3        10.5          180.          2.

NONB
  M1          1.3950  0.0149170000       IOD set for Zn2+ ion from Li et al.
JCTC, 2013, 9, 2733
  Y1          1.8240  0.1700             OPLS
  Y2          1.8240  0.1700             OPLS
  Y3          1.6612  0.2100             OPLS
  Y4          1.7683  0.1520             TIP3P water model
```

References

1. Lan, J.; Ge, J.; Yu, J.; Shan, S.; Zhou, H.; Fan, S.; Zhang, Q.; Shi, X.; Wang, Q.; Zhang, L.; Wang, X. Structure of the SARS-CoV-2 Spike Receptor-Binding Domain Bound to the ACE2 Receptor. *Nature* **2020,** *581*, 215-220.
2. Berman, H. M.; Westbrook, J.; Feng, Z.; Gilliland, G.; Bhat, T. N.; Weissig, H.; Shindyalov, I. N.; Bourne, P. E. The Protein Data Bank. *Nucleic Acids Res* **2000,** *28*, 235-42.
3. Anandakrishnan, R.; Aguilar, B.; Onufriev, A. V. H++ 3.0: Automating pK Prediction and the Preparation of Biomolecular Structures for Atomistic Molecular Modeling and Simulations. *Nucleic Acids Res.* **2012,** *40* (Web Server issue), W537-41.
4. Pengfei, L.; Kenneth, M. M. MCPB.py: A Python Based Metal Center Parameter Builder. *J. Chem. Inf. Model.* **2016,** *56*, 599-604.
5. Case, D. A.; Ben-Shalom, I. Y.; Brozell, S. R.; Cerutti, D. S.; Cheatham, T.E. III; Cruzeiro, V. W. D.; Darden, T. A.; Duke, R. E.; Ghoreishi, D.; Giambasu, G.; Giese, T.; Gilson, M. K.; Gohlke, H.; Goetz, A .W.; Greene, D.; Harris, R.; Homeyer, N.; Huang, Y.; Izadi, S.; Kovalenko, A.; *et al.* AMBER 2019, University of California, San Francisco, CA, USA. **2019**.
6. Gordon, M. S.; Schmidt, M. W. Advances in Electronic Structure Theory: GAMESS a Decade Later. In *Theory and Applications of Computational Chemistry*; Dykstra, C. E., Frenking, G., Kim, K. S., Scuseria, G. E., Eds.; Elsevier: Amsterdam, 2005; pp 1167-1189.
7. Maier, J. A.; Martinez, C.; Kasavajhala, K.; Wickstrom, L.; Hauser, K. E.; Simmerling, C. ff14SB: Improving the Accuracy of Protein Side Chain and Backbone Parameters from ff99SB. *J. Chem. Theory Comput.* **2015,** *11*, 3696-3713.
8. Kirschner, K. N.; Yongye, A. B.; Tschampel, S. M.; Gonzalez-Outeirino, J.; Daniels, C. R.; Foley, B. L.; Woods, R. J. GLYCAM06: A Generalizable Biomolecular Force Field. Carbohydrates. *J. Comput. Chem.* **2008,** *29*, 622-655.

9. Jorgensen, W. L.; Chandrasekhar, J.; Madura, J. D.; Impey, R. W.; Klein, M. L. Comparison of Simple Potential Functions for Simulating Liquid Water. *J. Chem. Phys.* **1983,** *79*, 926-935.
10. Berendsen, H. J. C.; Postma, J. P. M.; van Gunsteren, W. F.; DiNola, A.; Haak, J. R. Molecular Dynamics with Coupling to an External Bath. *J. Chem. Phys.* **1984,** *81*, 3684-3690.
11. Toukmaji, A.; Sagui, C.; Board, J.; Darden, T. Efficient Particle-Mesh Ewald Based Approach to Fixed and Induced Dipolar Interactions. *J. Chem. Phys.* **2000,** *113*, 10913–10927.
12. Loncharich, R. J.; Brooks, B. R.; Pastor, R. W. Langevin Dynamics of Peptides: the Frictional Dependence of Isomerization Rates of N-Acetylalanyl-N'-Methylamide. *Biopolymers* **1992,** *32*, 523–535.
13. Ryckaert, J.-P.; Ciccotti, G.; Berendsen, H. J. C. Numerical Integration of the Cartesian Equations of Motion of a System with Constraints: Molecular Dynamics of n-Alkanes. *J. Comput. Phys.* **1977,** *23*, 327-341.
14. Pettersen, E. F.; Goddard, T. D.; Huang, C. C.; Couch, G. S.; Greenblatt, D. M.; Meng, E. C.; Ferrin, T. E. UCSF Chimera - A Visualization System for Exploratory Research and Analysis. *J. Comput. Chem.* **2004,** *25*, 1605–1612.
15. Yan, Y.; Yang, M.; Ji, C. G.; Zhang, J. Z. H. Interaction Entropy for Computational Alanine Scanning. *J. Chem. Inf. Model*. **2017**, *57*, 1112-1122.
16. Sun, Z.; Yan, Y. N.; Yang, M.; Zhang, J. Z. Interaction Entropy for Protein-Protein Binding. *J. Chem. Phys.* **2017,** *146*, 124124.
17. Liu, X.; Peng, L.; Zhou, Y.; Zhang, Y.; Zhang, J. Z. H. Computational Alanine Scanning with Interaction Entropy for Protein–Ligand Binding Free Energies. *J. Chem. Theory Comput.* **2018,** *14*, 1772-1780.
18. Wang, E.; Sun, H.; Wang, J.; Wang, Z.; Liu, H.; Zhang, J. Z. H.; Hou, T. End-Point Binding Free Energy Calculation with MM/PBSA and MM/GBSA: Strategies and Applications in Drug Design. *Chem. Rev.* **2019,** *119*, 9478-9508.
19. Moreira, I. S.; Fernandes, P. A.; Ramos, M. J. Computational Alanine Scanning Mutagenesis - An Improved Methodological Approach. *J. Comput. Chem.* **2007**, *28*, 644-654.
20. Simões, I. C.; Costa, I. P.; Coimbra, J. T.; Ramos, M. J.; Fernandes, P. A. New Parameters for Higher Accuracy in the Computation of Binding Free Energy Differences upon Alanine Scanning Mutagenesis on Protein-Protein Interfaces. *J. Chem. Inf. Model.* **2017,** *57*, 60-72.
